# Hepatocellular Carcinoma Presenting With Inferior Vena Cava Tumor Thrombus Extending to the Right Atrium: A Case Report

**DOI:** 10.7759/cureus.9421

**Published:** 2020-07-27

**Authors:** Ragia Aly, Sachin Gupta, Ruby Gupta, Vinicius M Jorge, Ahmed Ebraheem

**Affiliations:** 1 Internal Medicine, Danbury Hospital, Danbury, USA; 2 Internal Medicine, Reading Hospital, West Reading, USA; 3 Hematology and Medical Oncology, William Beaumont Hospital, Royal Oak, USA; 4 Hematology and Medical Oncology, Albert Einstein Medical Center, Philadelphia, USA; 5 Hospital Medicine, Danbury Hospital, Danbury, USA

**Keywords:** hepatocellular carcinoma, tumor thrombus, inferior vena cava tumor thrombus, right atrium tumor thrombus, hepatitis c (hcv) infection, cirrhosis, hcc, lenvatinib

## Abstract

Hepatocellular carcinoma (HCC) is the most common primary liver tumor, and its incidence has been on the rise worldwide. It is a common cause of cancer-related death. HCC carries a poor prognosis and is challenging to manage, especially when diagnosed in advanced stages. We present a rare case of HCC in liver cirrhosis secondary to viral hepatitis C (HCV) infection, presenting with large tumor thrombus extending to the right atrium treated with lenvatinib.

## Introduction

Multiple types of primary malignant tumors can arise from the liver, namely, hepatocellular carcinoma (HCC), cholangiocarcinoma, hemangiosarcoma, angiosarcomas and hepatoblastoma, with HCC being the most common, accounting for more than three-fourth of liver primary malignant tumors [1]. The common risk factors for HCC include chronic viral hepatitis types B and C, alcoholic liver cirrhosis, non-alcoholic steatohepatitis (NASH), and obesity [2]. Patients presenting with advanced or metastatic HCC have a very poor prognosis [3]. HCC is one of the tumors commonly associated with intravascular invasion and tumor thrombus formation [4]. Patients who present with tumor thrombus have worse outcomes and limited treatment options [5]. Our patient presented with HCC associated with extensive intravascular invasion with tumor thrombus extending to the right atrium and possible lung metastasis.

## Case presentation

A 70-year-old man with a medical history significant for viral hepatitis C (HCV) infection and liver cirrhosis initially presented with scrotal swelling, decreased appetite, and unintentional 15 Ib weight loss over the past two months. He also complained of abdominal distention, constipation, dizziness, and dyspnea on exertion. He denied any other symptoms. His social history was significant for tobacco smoking and consumption of two to three alcoholic beverages per week. He had no history of IV drug use. His family history was noncontributory. On physical examination, he weighed 139 Ib with a BMI of 21 kg/m2. His temperature was 36.9 degrees Celsius; heart rate was 97 beats per minute, blood pressure was 118/69 mmHg. His exam was notable for distended abdomen with positive fluid thrill and scrotal edema. The remaining physical exam was unremarkable. His ECOG performance status was two (ambulatory and capable of all self-care but unable to carry out any work activity; up and about >50% of waking hours). His blood work was significant for mildly elevated total bilirubin of 2.2 mg/dL (normal range 0.1-1.2 mg/dL), elevated AST of 108 IU/L (normal range 8-48 IU/L), low albumin of 2.3 g/dL (normal range 3.4-5.4 g/dL). His blood counts were normal except for borderline platelet count 156 x 10^3/mcL (normal 150-400 x 10^3/mcL). His partial thromboplastin time (PTT) was mildly elevated as 38.7 seconds, prothrombin time (PT) elevated as 15.7 seconds, and INR was 1.2. His viral hepatitis profile showed positive Hepatitis C antibodies, and his quantitative HCV RNA PCR was 484000 IU/mL. His Alfa fetoprotein was markedly elevated at 26315.6 ng/mL (normal < 10 ng/mL). He was classified as Child Class B based on his Child-Pugh score of 9.

**Figure 1:**
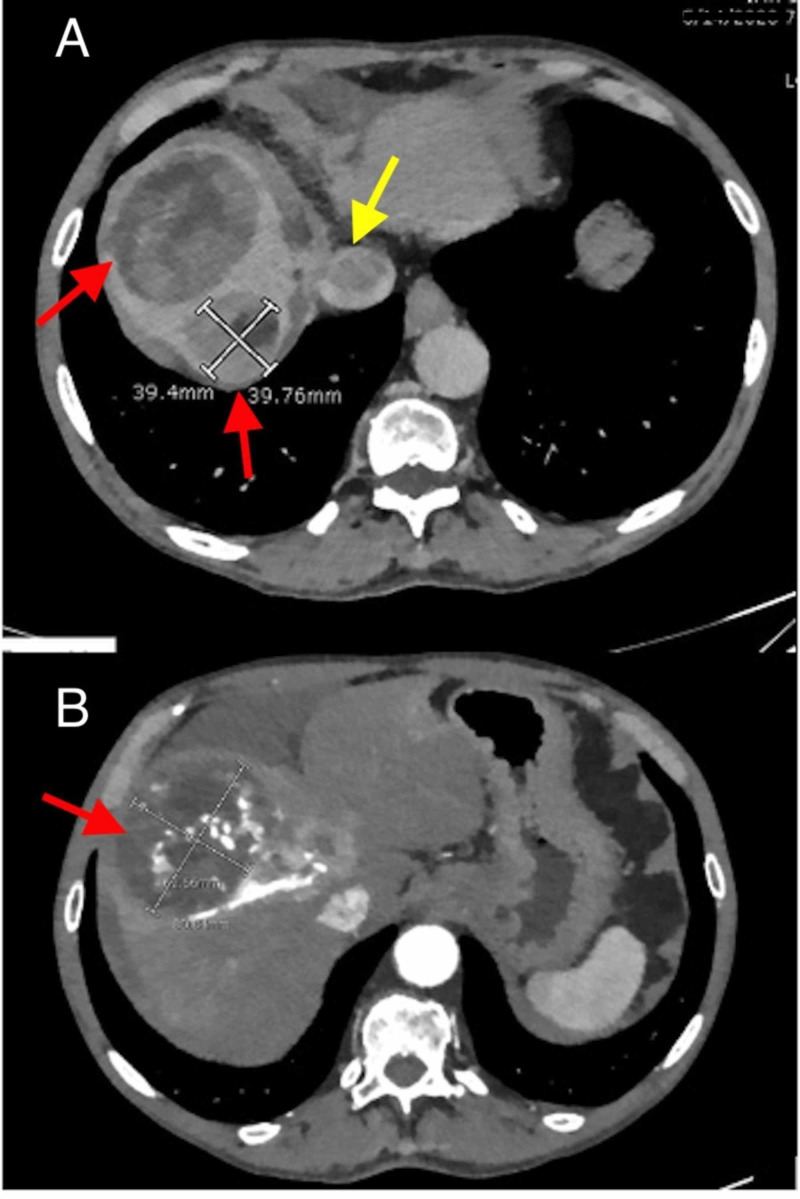
Abdominal CT scan with contrast. A: two hepatic observations consistent with HCC (red arrows), IVC tumor thrombus (yellow arrow). B: large hepatic observation (red arrow). HCC, hepatocellular carcinoma; IVC, inferior vena cava

An abdominal CT scan with IV contrast revealed two liver observations in the right hepatic lobe with LI-RADS score LR5 consistent with HCC, one was larger (8.1 cm) in segment VIII (Figure 1). The scan also demonstrates tumor thrombus in the right portal vein and the middle hepatic vein, which extends into the inferior vena cava (IVC) and the right atrium. The imaging also showed multiple additional smaller observations with LI-RADS score of LR4 and LR3. He also had a cirrhotic liver with evidence of portal hypertension and complex perihepatic ascites.

**Figure 2:**
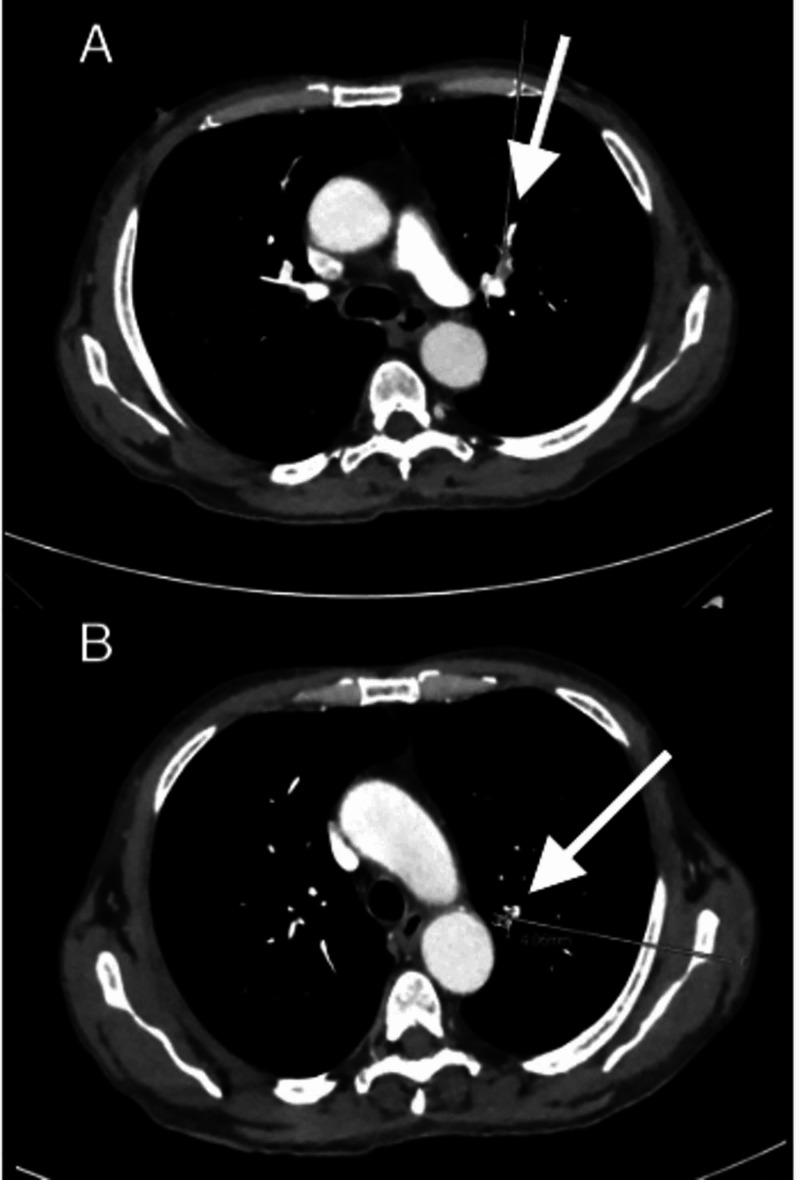
Chest CT scan with contrast. A: subsegmental pulmonary embolus (arrow). B: indeterminate pulmonary nodule (arrow).

A CT scan of the chest with IV contrast revealed acute pulmonary emboli in the segmental and subsegmental pulmonary arteries of the left upper lobe, subsegmental arteries of the left lower lobe, and proximal subsegmental arteries of the right lower lobe (Figure 2). He was also found to have bilateral pulmonary indeterminate micronodules concerning for metastatic lesions.

The patient was diagnosed with metastatic HCC with tumor thrombus extending to the IVC and right atrium. The patient was not found to be a candidate for any surgical intervention or locoregional therapy due to the presence of multiple liver masses, large tumor thrombus, high Child-Pugh score, and the possibility of lung metastases. He was started on palliative systemic therapy with lenvatinib 12 mg orally once daily according to the current National Comprehensive Cancer Network (NCCN) guidelines. He was prescribed low molecular weight heparin (LMWH) subcutaneous injections 60 mg/0.6 mL every 12 hours. The patient was seen in the clinic for follow up after two weeks. He did not report any adverse reactions. However, he did not want to continue the LMWH injections due to significant discomfort. The patient was switched to the direct-acting oral anticoagulant apixaban 5 mg twice daily.

## Discussion

Hepatocellular carcinoma is the second most common cause of cancer-related mortality worldwide [6]. HCC can grow for a period of time without causing any symptoms. Once patients are symptomatic, they usually have a locally advanced or metastatic disease, which carries a poor prognosis and high mortality [7]. Despite screening for HCC among high risk patients being recommended by the American Association for the Study of Liver Diseases, the NCCN and multiple other organizations, the process is not streamlined [8]. Currently, the US preventive task force does not include screening for HCC among its recommendations. Portal vein tumor thrombus is commonly found in patients with HCC. The presence of extensive thrombosis is considered a poor prognostic factor and an indicator of a more aggressive tumor and higher risk for recurrence and metastatic disease [9]. Less than 5% of patients with HCC present with tumor thrombus extending to the IVC and the right atrium. These patients have the added risk of developing cor pulmonale and pulmonary embolism, which can lead to sudden cardiac death [10]. Surgical removal of right atrial thrombus and inferior vena cava thrombus if successful carries the best outcomes for these patients; however, it is associated with increased risk, as it requires patients to be placed on cardiopulmonary bypass, which can only be done safely in few selected cases of HCC with low Child-Pugh scores [11].

A few studies and case reports described patients with tumor thrombus extending to the right atrium without metastatic disease who underwent locoregional treatment with improved outcomes and limited complications. Li et al. described a case of HCC with tumor thrombus, which was treated with transcatheter arterial chemoembolization (TACE) followed by percutaneous microwave ablation with favorable response [11]. In their retrospective study, Zhu et al. reported that 18 patients treated with TACE had improved overall survival and improved HCC associated symptoms such as ascites and improved liver function tests [12]. Our patient had multiple large hepatic lesions and suspected lung metastasis; he also had elevated Child-Pugh score. He was not a candidate for locoregional therapy or surgical resection of his thrombus or tumor. His only option was palliative systemic therapy. The NCCN recommends first-line therapy with lenvatinib, a multiple kinase inhibitor that works against vascular endothelial growth factor receptors 1, 2, and 3 kinases [13]. This drug was FDA approved in 2018 as a first-line therapy for the treatment of HCC after it had shown noninferiority to sorafenib in overall survival and a statistically significant longer progression-free survival in patients with advanced HCC [14]. The immune checkpoint inhibitors atezolizumab and bevacizumab are also available as an option for first-line systemic therapy in HCC. There is currently an increasing role for immune checkpoint inhibitors in treating HCC and multiple ongoing clinical trials to study their outcomes [15].

## Conclusions

Patients with HCC and liver cirrhosis who present in advanced disease stage or with large tumor thrombus have limited treatment options and poor outcomes. This case adds to the limited number of reported cases of HCC with IVC tumor thrombus extending to the right atrium. More clinical trials and new treatments are needed to improve the outcomes in patients with advanced HCC. Improving and streamlining HCC screening among high-risk patients may result in early diagnosis and better patient outcomes.
